# Oral and sublingual immunotherapy for food allergy

**DOI:** 10.1186/1939-4551-7-35

**Published:** 2014-12-08

**Authors:** Uyenphuong H Le, A Wesley Burks

**Affiliations:** Department of Pediatrics, University of North Carolina at Chapel Hill, Chapel Hill NC 260 MacNider Hall, CB 7220, Chapel Hill, NC 27599-7220 USA

**Keywords:** Food allergy, Oral immunotherapy, Sublingual immunotherapy, Desensitization, Tolerance

## Abstract

IgE-mediated food allergy is a potentially life-threatening allergic disease with an increase in prevalence in developed countries over the past 15 years. Currently, there are no approved forms of therapy and the standard of care is dietary restriction and ready access to emergency medications, such as self-injectable epinephrine and antihistamines. Allergen-specific modalities of treatment currently being studied include oral immunotherapy (OIT) and sublingual immunotherapy (SLIT). Both forms demonstrate the ability to desensitize patients to a variety of specific food allergens and show great promise. However, more research is needed to evaluate the safety and efficacy of OIT and SLIT prior to routine use in clinical practice.

## Introduction

Food allergy is a major public health concern that affects approximately 8% of US children [1]. The most common food allergens that elicit IgE-mediated reactions include milk, egg, peanut, tree nuts, wheat, soy, fish and shellfish [2]. Of these, peanut allergy is the most common cause of anaphylaxis in children presenting to the emergency department, as well as the most common cause of fatal food anaphylaxis [3, 4]. The prevalence of peanut allergy has tripled from 0.4% to 1.4% from 1997 to 2008 [5, 6]. Approximately 85% of children allergic to foods such as cow’s milk, egg, wheat and soy will outgrow their allergy, whereas 80-85% of children allergic to peanut, tree nuts, fish and shellfish will not [7].

There is currently no approved treatment or disease-modifying therapy for the routine management of patients with food allergies. The present standard of care is strict dietary avoidance of appropriately-diagnosed food allergens and ready access to emergency medications, such as self-injectable epinephrine and antihistamines [2]. Despite parent and patient vigilance with food allergen avoidance, accidental exposures resulting in clinical symptoms do occur [8]. As a result, patients and their families experience significant psychosocial burden and diminished health-related quality of life [9, 10]. Recent efforts have focused on developing safe and effective therapies for patients with food allergies, with the most active research involving oral and sublingual immunotherapy (OIT and SLIT).

## Mechanism for the development of food allergy

Oral tolerance is the process by which previously encountered proteins exposed to the gastrointestinal tract are tolerated through the suppression of cellular or humoral immune responses [11]. This suppression occurs through a number of mechanisms including the production of regulatory T cells (Tregs), the deletion of antigen-specific T cells, or the induction of anergy in antigen-specific T cells [11, 12]. Food hypersensitivity is thought to result from either the failure to establish or the breakdown of existing oral tolerance [12]. Essentially, food allergy starts with an initial sensitization event to the food protein; however, the route and timing by which sensitization occurs remains unclear. Patients with a predisposition for food allergy develop a T helper (Th)2-predominant immune response. Th2 cells secrete cytokines including interleukin (IL)-4, IL-5 and IL-13, which stimulate B cells to produce allergen-specific IgE. These IgE antibodies bind to the surface of mast cells and basophils by high affinity receptors and cross-link upon re-exposure to the protein allergen, releasing mediators such as histamine, leukotrienes, cytokines and prostaglandins. These mediators lead to symptoms of allergic reactions [13].

## Allergen immunotherapy

Allergen immunotherapy is a form of treatment that involves administering gradually increasing doses of allergen over time to induce immunologic changes. There are two possible immune states that can be achieved through food allergen immunotherapy: desensitization and tolerance. Desensitization occurs when daily allergen exposure increases the threshold of clinical reactivity to the food. Patients are therefore able to tolerate more food protein during an oral food challenge while on treatment. When dosing is stopped or interrupted, the protection is lost or reduced. However, the ultimate goal of allergen immunotherapy is tolerance, which is the ability to ingest the food without allergic symptoms after discontinuation of the therapy. Immunologic changes during immunotherapy show a shift away from a Th2 profile with decrease reactivity of mast cells and basophils, increase Treg production, increase food-specific IgG4 antibodies and eventual decrease in food-specific IgE antibodies [7].

Current protocols in food immunotherapy involving OIT and SLIT typically comprise of 3 phases: (1) an initial modified dose escalation or modified rush desensitization that takes place over 1-2 days with 6-8 doses of the allergen given, (2) a build-up phase that consists of weekly to biweekly dose escalations performed over 6-12 months and (3) a maintenance phase with daily home dosing that occurs over months to years. Oral food challenges (OFC) are used to test clinical reactivity while on treatment (desensitization) and while off therapy but still on diet restriction (tolerance) [7, 14]. The initial desensitization and dose escalations, as well as OFCs, are performed in a supervised clinical setting while maintenance dosing is carried out at home.

## OIT

OIT involves the daily administration of food allergen (milligrams to grams) mixed with a food vehicle in gradually increasing doses over months to years. OIT has been studied in several uncontrolled clinical studies for more than a decade involving mainly milk, egg and peanut allergies. Recent trials have provided invaluable efficacy and safety data as well as compelling evidence that OIT frequently induces desensitization, and possibly even tolerance, in patients with food allergies (Table 1).

**Table 1:** **Summary of selected studies reviewed**

Study	Subjects	Age of subjects	Baseline OFC	Goal maintenance dose & duration of IT	Clinical outcome/Desensitization n (%)	Drop outs	Tolerance n (%)	Immunologic changes	Safety data
**Burks et al,** [15] *Randomized, double-blind, placebo-controlled clinical trial. Egg OIT*	N = 55	5-11 yrs.	No	Dose = 2000 mg	30 (75%) passed 10 g OFC and desensitized at 22 mos. of OIT	8	11 (28%) at 23 mos. – off 4-6 wks.	↑egg white-specific IgG4; ↓egg white-specific IgE and basophil activation	No severe adverse events. 78% of active OIT children had oral or pharyngeal AEs vs. 20% in placebo group
				Duration = 22 mos.					
**Varshney et al,** [16] *Randomized, placebo-controlled clinical trial. Peanut OIT*	N = 28	1-16 yrs.	No	Dose = 4000 mg	16 (84%) passed 5 g OFC	3	Not assessed	No change in IgE; ↑IgG4 and peanut-specific FoxP3 Tregs; ↓IL-5 and IL-13; ↓skin prick tests	Initial escalation: 9 (47%) of 19 OIT subjects had AEs with 2 requiring epinephrine
				Duration = 48 wks.					Build-up doses: OIT subjects experienced AEs with 1.2% of 407 doses, no epinephrine required
**Vickery et al,** [17] *Open-label, uncontrolled trial. Peanut OIT*	N = 39	1-16 yrs.	No	Initial pilot OIT trial: Dose = 300 mg	Initial pilot OIT trial: 27 (93%) of 29 passed 3.9 g OFC and considered desensitized	15	Continued OIT trial: 12 (50%) of 24 achieved sustained unresponsiveness (treatment successes) – off 4 weeks	↓skin prick tests, ↓peanut, Ara h 1, & Ara h 2 IgE, ↓peanut IgE/total IgE ratio, no change in peanut IgG4 or functional activity	Initial pilot OIT trial: 92% with AE during 1-day dose escalation; 46% of build-up doses elicited symptoms
				Duration = 8 mos.					
				Continued OIT trial: Dose = 4000 mg	Continued OIT trial: 24 (100%) of 24 passed 5 g desensitization OFC				Continued OIT trial: No AEs reported by treatment successes with peanut exposure vs. 3 (14%) of treatment failures reported mild reactions
				Duration = 5 yrs.					
**Fleischer et al,** [30] *Randomized, double-blind, placebo-controlled clinical trial. Peanut SLIT*	N = 40	12-37 yrs.	Yes	Dose = 165-1386 mcg	14 (70%) in active group vs. 3 (15%) in placebo group were responders	10	Not assessed	No change in peanut-specific IgE; ↑IgG4	First phase: AEs with 40.1% of 5825 peanut SLIT doses including 1 treated with epinephrine vs. 0.6% of 6029 placebo doses
				Dose for cross-over group = 3696 mcg					Crossover High dose group: 33.3% of 5030 doses had AEs
				Duration = 44 wks.					
**Keet et al,** [29] *Randomized open-label clinical trial. Combined cow’s milk SLIT/OIT*	N = 30	6-17 yrs.	Yes	SLIT dose =7 mg	1 (10%) SLIT	2	1 SLIT and 8 combined OIT subjects deemed tolerant	↑cow’s milk-specific IgG4 in all groups; ↓specific-IgE and basophil response in combined OIT	Symptoms with 1802 (29%) of 6246 SLIT doses and 2402 (23%) of 10,645 OIT doses. OIT had significantly more multisystem, upper respiratory tract, gastro-intestinal, lower respiratory tract symptoms, more need for B-agonist and antihistamines. Epinephrine 2× in SLIT and 4× in OIT.
				OITB dose =1000 mg	6 (60%) SLIT/OITB				
				OITA dose =2000 mg	8 (80%) SLIT/OITA passed 8 g OFC				
				SLIT duration = 74 wks.					
				OIT duration = 86 wks.					

In 2012, Burks et al. published the first multicenter, randomized, double-blind, placebo-controlled trial of egg OIT [15]. The study was designed to evaluate the clinical effect of egg OIT on desensitization and “sustained unresponsiveness,” the ability to consume 10 g of egg-white powder after 22 months of OIT and subsequent avoidance of egg for 4 to 6 weeks. Fifty-five children were enrolled with 40 subjects receiving egg OIT and 15 placebos. Subjects underwent a 1-day dose escalation, and a build-up phase to a goal maintenance dose of 2000 mg. After 10 months, subjects underwent the first 5 g OFC where 22 (55%) active subjects passed (desensitized) versus none in the placebo group. The study was unblinded at this point and the active treatment group was continued on maintenance OIT until a 22-month 10 g OFC, during which 30 (75%) of the active group passed (desensitized) compared to 0 (0%) of the placebo group. Active subjects who passed the 22-month OFC were taken off OIT for 4 weeks and returned for another 10 g OFC to determine sustained unresponsiveness (tolerance). Eleven (28%) active subjects passed and continued to incorporate egg into their diet without symptoms. Symptoms occurred with 25% of the total doses taken by the actively treated subjects compared to 3.9% of placebo doses. No serious therapy-related adverse reactions were reported. Egg white-specific IgG4 was higher for subjects who passed OFCs at 10, 22 and 24 months than for those who did not. Higher IgG4 levels at 10 months also suggested ability to pass OFCs at all 3 time points. Egg white-specific IgE and basophil activation levels were lower at 10 months for subjects who passed the 22-month OFC compared to those who failed.

In 2011, Varshney et al. published the first randomized, double-blind, placebo-controlled study to date, which conclusively demonstrated that peanut OIT induces desensitization and immune modulation [16]. Twenty-eight children were enrolled. However, 3 subjects withdrew early leaving 16 subjects in the active treatment group and 9 in the placebo group. The study protocol included 1-day initial escalation, 44-week build-up and 4-week maintenance phases (goal maintenance dose 4000 mg peanut protein) followed by a 5 g OFC at about 1 year. All 16 (100%) subjects receiving OIT passed the 5 g OFC compared to the median cumulative dose tolerated by the placebo group of 280 mg. The peanut OIT was well tolerated with subjects experiencing symptoms after only 1.2% of build-up doses. No active subjects required epinephrine with dose escalation visits or home doses. Peanut OIT subjects showed decrease IL-5 and IL-13, increase in peanut-specific IgG4 and peanut-specific FoxP3 Tregs, but no significant change in peanut-specific IgE at the time of OFC.

Subsequently, the first study to demonstrate clinical tolerance, or sustain unresponsiveness, after peanut OIT was recently published by Vickery et al. [17], who reported end-of-study results from an initial pilot trial of peanut OIT that was published in 2009 [18]. Twenty-four of 39 subjects originally enrolled in the pilot trial at 2 US centers were recruited to continue this OIT protocol. The maximum daily maintenance dose was 4000 mg. Subjects were treated for either a maximum duration of 5 years or once specific criteria were met, such as peanut IgE less than 2 kU/L or less than 15 kU/L with peanut skin prick test response less than 5 mm and no peanut-related reactions in the previous 6 months. Subjects then underwent two 5 g DBPCFC. The first was a desensitization OFC performed to assess clinical reactivity while receiving treatment and the second was to evaluate for sustained unresponsiveness after stopping OIT for 4 weeks. Twelve (50%) of the 24 subjects consumed 5 g of peanut protein and passed the open oral feeding of peanut butter without symptoms. These subjects were considered to have achieved sustained unresponsiveness and classified as treatment successes. Immunologically, they had smaller skin test results as well as lower peanut-specific IgE, Ara h 1, Ara h 2 levels and peanut-specific IgE/total IgE ratios. However, there were no between-group differences in peanut IgG4 levels or Treg cell numbers. Although this study lacked randomization and a placebo control group, it describes for the first time that immune tolerance, or sustained unresponsiveness, among children with peanut allergy treated with OIT is possible.

## SLIT

SLIT involves the administration of small drops of allergen extract (micrograms to milligrams) under the tongue, which is then eventually spit or swallowed. Doses are approximately 1000-times less than OIT doses, but SLIT protocols include similar escalation and maintenance dosing [14, 19]. The mechanism of action involves allergen interaction with protolerogenic Langerhans cells in the oral mucosa, resulting in suppression of the allergic response [20]. SLIT has been shown to be effective for other atopic diseases such as asthma and allergic rhinoconjunctivitis [21, 22]. A number of studies involving SLIT and a variety of food allergens, including kiwi [23], hazelnut [24, 25], milk [26], peach [27] and peanut allergies have been promising. Thus far, available evidence suggests that SLIT is less effective than OIT of inducing desensitization, but has a better safety profile given the low dose of peanut allergen required for treatment [28, 29].

Fleischer et al. recently published results from a randomized, double-blind, placebo-controlled multicenter trial of peanut SLIT with a crossover design in which 40 subjects, adolescents and young adults, were enrolled after baseline OFC of up to 2 g of peanut powder [30]. Subjects were randomized 1:1 across 5 sites to receive either daily peanut or placebo SLIT. At 44 weeks, a 5 g OFC was performed, followed by unblinding of the protocol. Placebo-treated subjects were then crossed over to receive higher dose peanut SLIT, followed by a subsequent 5 g OFC after 44 weeks on SLIT. OFCs on 44 weeks of SLIT were compared to baseline OFCs from both groups and subjects were considered responders if they successfully consumed 5 g or at least 10-fold more peanut powder than at baseline. Fourteen (70%) active subjects were considered responders compared to 3 (15%) placebo subjects. In peanut SLIT responders, the median successfully consumed dose increased from 3.5 to 496 mg after 44 weeks on therapy. When challenged again after 68 weeks of SLIT, the median dose consumed again significantly increased to 996 mg. This study clearly showed that peanut SLIT can safely induce a desensitized state in a majority of subjects compared with placebo and that the length of therapy can significantly affect the amount of allergen consumed.

## Combined SLIT/OIT

In the first study to compare SLIT with OIT, Keet el al. published the results of a randomized clinical trial of cow’s milk allergy in 30 children [29]. After entry DBPCFC, all subjects were treated with SLIT for 4 weeks. They were then randomized equally into 3 groups: (1) continued SLIT escalation to 7 mg daily, (2) cross over with OIT to 1000 mg (OITB), or (3) cross over with OIT to 2000 mg (OITA). Subjects were challenged with 8 g milk protein after 12 and 60 weeks of therapy. If they passed the 60-week challenge, therapy was stopped and challenges were repeated 1 and 6 weeks later to assess for tolerance.

After therapy, 1 (10%) in the SLIT group, 6 (60%) in the SLIT/OITB group and 8 (80%) in the SLIT/OITA group passed the 8 g OFC. However, the food challenge threshold increased in all subjects who completed the full maintenance period: 40-fold for SLIT/SLIT, 159-fold for SLIT/OITB and 54-fold for SLIT/OITA groups. After 1 week of avoidance, 2 subjects in the SLIT/OITB group reacted during challenge. After 6 weeks off therapy, an additional subject in the SLIT/OITB group and 3 subjects in the SLIT/OITA group failed the OFC. Therefore, this left 1 in the SLIT/SLIT and 8 in the combined SLIT/OIT groups who were considered tolerant.

Adverse reactions occurred more frequently with SLIT (29%) than OIT (23%) doses; however, while SLIT caused more mild symptoms such as oral pruritus, OIT doses caused more multisystem, gastrointestinal, upper and lower respiratory tract symptoms, as well as increased need for β-agonist and antihistamine treatment. Mechanistic studies revealed increased cow’s milk-specific IgG4 in all groups, but decreased specific-IgE and basophil histamine release only in the combined OIT subjects when compared to baseline. The study showed that OIT was more effective than SLIT alone in inducing desensitization to cow's milk, but not without more systemic side effects. While a handful of subjects achieved clinical tolerance after 1 and 6 weeks of avoidance, it is still unclear how long the clinical effects of immunotherapy lasts once exposure is stopped.

## Food immunotherapy in clinical practice

Recent studies, including those mentioned above, show promising data for the use of immunotherapy in food allergic patients. However, therapies such as OIT and SLIT are not recommended for routine clinical practice and the current standard of care in the treatment of food allergies is allergen avoidance and ready access to self-injectable epinephrine [31]. Despite these recommendations, a recent article summarizes the results of a retrospective chart review of patients treated with peanut OIT in 5 different practices: 4 office-based practices in the United States and 1 hospital-based practice in Israel [32]. The authors report a total of 352 treated patients who received 240,351 doses of peanut, peanut butter, or peanut flour, and experienced 95 reactions that required epinephrine administration. The study cites a success rate of 85% based on the number of patients who achieved goal maintenance dose and a reaction rate of 0.7 per 1000 doses during escalation and 0.2 of 1000 doses during maintenance. It should be noted that the OIT methods used at each site were locally developed and had considerable variability, including maintenance doses ranging from 415 to 8000 mg, differences in selection criteria for enrolling patients, definition for mild reactions and criteria for administering epinephrine. Despite these differences, the authors conclude that peanut OIT may be a suitable therapy when managed by qualified allergists/immunologists.

However, several recent systematic reviews and meta-analyses do not support this notion and conclude that “there is insufficient evidence in terms of long-term effectiveness, safety, and cost-effectiveness of peanut OIT to recommend its routine use in clinical practice [33, 34].” At this time, variations in immunotherapy protocols, such as study product used, starting and ending doses, study schedule, blinding, use of placebo, selection of study subjects and reporting of adverse reactions, make direct comparisons and evaluation of true efficacy and safety of OIT and SLIT difficult [35, 36]. Experts in the field strongly recommend that these experimental therapies continue to be administered under the oversight of institutional review boards and the US Food and Drug Administration as food OIT remains in a state of equipoise [37].

## Conclusions

Food allergy is an increasingly prevalent disorder in the United States and other Westernized countries with no definitive cure or approved treatment. Patients living with food allergies are at risk of accidental ingestions daily that can result in potentially life-threatening reactions. Over the past decade, there has been resurgence in interest and an increase number of clinical trials to evaluate immunotherapy options for food allergy, particularly OIT and SLIT. Several studies have demonstrated the ability of OIT and SLIT to induce desensitization, in which patients are able to tolerate the ingestion of more food allergen while on treatment, and immunologic changes with ongoing therapy. However, concerns and questions still remain regarding the allergic side effects and the development of immune tolerance with these therapies, the ultimate goal of allergen immunotherapy in which patients tolerate the ingestion of food off treatment. Further research is needed to address the safety and efficacy of OIT and SLIT for long-term use.

## Acknowledgements

Support for the dissemination of the WAO Immunotherapy and Biologics Online Monograph is provided by the following sponsors: Circassia, Boehringer-Ingleheim, and ORA Inc.

## Abbreviations

IgE: Immunoglobulin E
OIT: Oral immunotherapy
SLIT: Sublingual immunotherapy
Tregs: Regulatory T cells
Th: T helper
IL: Interleukin
OFC: Oral food challenge
DBPCFC: Double-blind, placebo-controlled food challenge.
